# Evaluation of the learning curve of laparoscopic choledochal cyst excision and Roux-en-Y hepaticojejunostomy in children: CUSUM analysis of a single surgeon’s experience

**DOI:** 10.1007/s00464-016-5032-5

**Published:** 2016-06-23

**Authors:** Zhe Wen, Huiying Liang, Jiankun Liang, Qifeng Liang, Huimin Xia

**Affiliations:** 1 Department of Pediatric Surgery, Guangzhou Women and Children’s Medical Center, Guangzhou Medical University, 9 Jinsui Road, Tianhe District, Guangzhou, 510623 China; 2 Institute of Pediatrics, Guangzhou Women and Children’s Medical Center, Guangzhou Medical University, Guangzhou, China

**Keywords:** Choledochal cyst, Learning curve, Laparoscopy

## Abstract

**Introduction:**

Laparoscopic cyst excision and Roux-en-Y hepaticojejunostomy is gaining popularity as a treatment for choledochal cyst (CDC) in children. However, the learning curve for this challenging laparoscopic procedure has not been addressed. The aim of this study is to determine the characteristics of the learning curve of this procedure. This may guide the training in institutions currently not using this technique.

**Methods:**

A prospectively collected database comprising all medical records of the first 104 consecutive patients undergoing laparoscopic CDC excision and Roux-en-Y hepaticojejunostomy performed by one surgeon was studied. Multifactorial linear/logistic regression analysis was performed to identify patient-, surgeon-, and procedure-related factors associated with operating times, rates of adverse event, and length of postoperative stay.

**Results:**

Cumulative sum analysis demonstrated a learning curve for laparoscopic choledochal cyst excision of 37 cases. Comparing the early with the late experiences (37 vs. 67 cases), the surgeon-specific outcomes significantly improved in terms of operating times (352 vs. 240 min; *P* < 0.001), postoperative complication rate (13.5 vs. 1.5 %; *P* = 0.02), and the length of hospital stay (9.4 vs. 7.8 days; *P* = 0.01). After multivariate analyses, independent predictors of operating times included the completion of the learning curve (CLC) (OR 0.68, 95 % CI 0.63–0.73) and adhesion score (OR_middle_ 1.25, 95 % CI 1.08–1.45; OR_high_ 1.40, 95 % CI 1.20–1.62; compared with the low score); significant predictors of perioperative adverse outcomes were CLC (OR 0.07, 95 % CI 0.02–0.34) and comorbidities prior to the surgery (OR 30.65, 95 % CI 1.71–549.63). The independent predictors of length of postoperative stay included CLC, preoperative comorbidities, and perioperative adverse events.

**Conclusions:**

CLC for laparoscopic choledochal cyst excision is 37 cases. After CLC, not only the operative time is reduced, the complications, adverse results, and the length of hospital stay all decreased significantly. The learning curve can be used as the basis for performance guiding the training.

Choledochal cyst (CDC) is a rare disease of the biliary tree among the western populations with an incidence of 1 in 13,000–15,000. However, it is not as rare in East Asian nations with an incidence as high as 1 in 1000 [1]. The classification system of choledochal cysts is based on the site of the cyst or dilatation, and it currently includes 5 major types, with Ia, Ic, and IVa being the most common types [2]. More than 2/3 patients with choledochal cysts have symptoms before 10 years of age, while it is rare to be asymptomatic until adulthood [3].

Total cyst excision and Roux-en-Y hepaticojejunostomy is the standard procedure for choledochal cyst [4]. Comparing with open procedure, laparoscopic procedure has been proven to have shorter hospital stay and lower morbidity of anastomotic stenosis, bile leakage, intrahepatic stone formation, cholangitis, pancreatic leak, intestinal obstruction, and re-operation [5–7]. Thus, as a safe, efficacious, and minimally invasive procedure, laparoscopic cyst excision and Roux-en-Y hepaticojejunostomy has become a common procedure for pediatric choledochal cyst in many medical centers [8].

However, conversions and complications are frequent especially in the early stage of the laparoscopic series, even for those who are well experienced in open surgical techniques. For example, Ure et al. [9] presented their experience with a first series of 11 patients and found that the operation was converted to open surgery in two patients, biliary leakage occurred in one patient, and an open laparotomy was conducted for postoperative recurrent cholangitis in another patient 3 months after operation. Similar results were also reported by Chokshi et al. [10]. With increased experience, the incidence of adverse events is reduced. For example, in Liem’s series of 400 cases of laparoscopic choledochal cyst excision, all of the bile leakage and abdominal fluid collection only occurred in the first 2 years [11]. All these results in turn suggest that in the case of choledochal cyst excision, the learning of the complex laparoscopic surgery in children is a stepwise process and quite a number of procedures are required before the technique can be safely performed.

Thus, a logical question arises as to what is a reasonable number of procedures an individual surgeon has to perform to achieve satisfactory outcome results? For laparoscopic cyst excision and Roux-en-Y hepaticojejunostomy to continue gaining popularity and widespread application, a learning curve needs to be defined to guide the training. However, to the best of our knowledge, there has been no publication addressing the learning curve of laparoscopic cyst excision in children. Hence, we sought to establish a learning curve for the surgical steps of the laparoscopic procedure in children with CDCs, as performed by a single surgeon during his first 104 procedures.

## Patients and methods

### Design, population, and data collection

A retrospective study was performed on prospectively collected data from the first 104 consecutive children with CDCs who underwent minimally invasive laparoscopic cyst excision and Roux-en-Y hepaticojejunostomy from December 2010 through December 2014 at Guangzhou Women and Children’s Medical Center, China. The operations were performed by a single surgeon who was trained in pediatric minimal invasive surgery. The previous experience included laparoscopic appendectomy, laparoscopic-assisted transanal endorectal pull-through for Hirschsprung’s Disease, laparoscopic Ladd’s procedure, etc. The current study has been approved by the Institutional Review Board of the center. Prospective database was collected by investigators and the study coordinators through patients’ guardians and the referring physicians. The database provided a comprehensive dataset comprising of patient demographic characteristics, preoperative assessment, surgical treatment, postoperative course, intraoperative and postoperative complications, conversion to open procedure, and postoperative length of stay in hospital.

These selected patients represented 88.9 % of the total 117 choledochal cyst cases managed by this surgeon in the study period. The decision for open procedure primarily was based on the following: a secondary operation after the initial external drainage or the cystoenterostomy, the patients’ preference or the counter-indication for radical cyst excision.

### Details on the procedure

The technique of laparoscopic choledochal cyst excision has been described in our previous publication [12]. Briefly, (1) under general anesthesia, the patient was intubated and placed in reverse Trendelenburg position. (2) Four-site procedure was used as the trocars were located at middle of the umbilicus, right hypochondrium, right side of the abdomen, and left hypochondrium, respectively. (3) A monopolar electrocautery hook was used to dissect the choledochal cyst and the gallbladder. The cyst was dissected down to the distal tapered end of the common bile duct, and it was then ligated. The upper part of the cyst was further dissected up to the common hepatic duct and then removed at this level. When severe adhesion around the cyst was encountered, bipolar coagulation was used for dissecting. (4) A Roux-en-Y anastomosis was constructed by exteriorization of the small bowel via the enlarged umbilical trocar port. A retrocolic end-to-side hepaticojejunostomy was carried out laparoscopically. To minimize the biliary contamination of peritoneum, our procedure was modified later in the series by completing the jejunojejunostomy before the cyst excision. (5) Draining tube was indicated only in selected cases. (6) When common hepatic duct stenosis was encountered in the Todani-IVa type cases, the stenosis was resected or a ductoplasty was performed by a longitudinal incision on the anterior wall and followed by additional cholangioenterostomy.

### Study endpoint and risk factors

The primary endpoint was defined as the number of operations required to decrease operative times and complication rates to a steady level. Secondary endpoints included operating time, perioperative adverse events, and postoperative length of stay in hospital. The total operative time was defined as the time interval from skin incision to skin closure. Cyst excision time was defined as the time from dissection of the gallbladder/choledochal cyst to excision of the cyst and ductoplasty of the common hepatic bile duct if needed. Anastomosis time was defined as the time from incision of the jejunal wall of the Roux-en-Y loop to the completion of the hepaticojejunostomy.

Patient-specific factors included age, gender, length of history, comorbidity, whether or not the jaundice was resolved by conservative treatment, Todani’s classification type, and size of the cyst. The comorbidities in this study include two cases of accessory hepatic duct and one paraduodenal hernia. Intraoperative factor was mainly the extent of adhesion. Surgeon-specific factor, or the operative experience, was represented by surgeon’s case sequence number. Estimated blood loss was recorded after reconciling surgical and anesthesia records. Adhesive tenacity was classified into 3 degrees: mild, moderate, and severe.

### Statistical analyses

The cumulative sum (CUSUM) technique for assessment of the learning curve was applied to explore the relationship between operation time and sequence number of the laparoscopic procedure [13]. The CUSUM series was defined as Sn = ∑(*X*_i_ − *X*_0_), where *X*_i_ was an individual measurement and *X*_0_ was a predetermined reference level and was set as the mean operative time for all the cases here. Sn was plotted against the sequence of operations. Cutoff values were chosen according to the points of downward inflection revealed by the plots. The CUSUM was used to analyze the overall operation time, excision time, and anastomotic time, respectively.

The patients were divided into two groups according to the cutoff point of CUSUM score: group A (≤cutoff value) representing the early-experience group and group B (>cutoff value) the late-experience group. Variables included the proportions, means, or medians with variability estimates in the form of standard deviations (SD) and interquartile ranges (IQR), as appropriate. Chi-square test or Fisher’s exact test was used to compare the distribution of categorical variables between groups. Continuous variables were analyzed using Student’s *t* test or ANOVA. Multivariate analyses were performed using logistic regression model for the adverse outcome and linear regression model for the lengths of the operation and hospital stay, respectively. Statistical significance was defined as a two-sided *P* value <0.05. Statistical analysis was performed using SPSS version 19.0 (IBM SPSS Statistics for Windows, IBM Corporation, Somers, NY) unless otherwise specified.

## Results

A total of 104 patients (18 boys and 86 girls) underwent laparoscopic choledochal cyst excision surgery during the study period. The average age, disease duration, and cyst size in the series were 35.9 ± 24.3 months, 226.8 ± 345.2 days, and 3.4 ± 3.0 cm, respectively (Table 1).

**Table 1:** Characteristics stratified by the completion of the learning curve (CLC) cutoff

Variables	Total (*n* = 104)	CLC cutoff		*t*/*χ* ^2^	*P*
		Group A (*n* = 37)	Group B (*n* = 67)		
*Preoperative characteristics*					
Sex, no. (%)					
Male	18/104 (17.3)	7/37 (18.9)	11/67 (16.4)	0.10	0.75
Female	86/104 (82.7)	30/37 (81.1)	56/67 (83.6)		
Age, mean (SD), months	35.9 (24.3)	40.7 (26.1)	33.2 (23.0)	1.52	0.13
Comorbidity, no. (%)					
None	101/104 (97.1)	37/37 (100)	64/67 (95.5)	–^a^	0.55
One or more	3/104 (2.9)	0/37 (0)	3/67 (4.5)		
Disease duration, mean (SD), days	226.8 (345.2)	291.8 (449.1)	190.9 (268.8)	2.06	0.16
Type of cyst, no. (%)					
1a	64/104 (61.5)	23/37 (62.2)	41/67 (61.2)		
1c	25/104 (24.0)	8/37 (21.6)	17/67 (25.4)	0.27	0.87
4a	15/104 (14.4)	6/37 (16.2)	9/67 (13.4)		
Size of cyst, mean (SD), cm	3.4 (3.0)	3.3 (2.1)	3.4 (3.4)	0.09	0.93
Abdominal pain, no. (%)					
No	28/104 (26.9)	7/37 (18.9)	21/67 (31.3)	1.87	0.17
Yes	76/104 (73.1)	30/37 (81.1)	46/67 (68.7)		
Jaundice, no. (%)					
No	75/104 (72.1)	30/37 (81.1)	45/67 (67.2)	2.30	0.13
Yes	29/104 (27.9)	7/37 (18.9)	22/67 (32.8)		
Jaundice subsided, no. (%)					
No	13/104 (12.5)	4/37 (10.8)	9/67 (13.4)	0.15	0.70
Yes	91/104 (87.5)	33/37 (89.2)	58/67 (86.6)		
*Intraoperative results*					
Operation time, mean (SD), min	278.5 (81.7)	352.2 (80.5)	240.5 (50.6)	8.37	<0.001
Excision time, mean (SD), min	117.6 (47.7)	144.8 (60.4)	102.4 (29.9)	4.77	<0.001
Anastomosis time, mean (SD), min	50.9 (27.2)	74.1 (29.6)	38.0 (14.2)	8.36	<0.001
Blood loss, mean (SD), ml	12.0 (18.9)	14.2 (18.0)	10.7 (19.4)	0.88	0.38
Adhesion score, no. (%)					
Low	48/104 (46.2)	17/37 (45.9)	31/67 (46.3)	2.50	0.29
Middle	23/104 (22.1)	11/37 (29.7)	12/67 (17.9)		
High	33/104 (31.7)	9/37 (24.3)	24/67 (35.8)		
Transfusion, no. (%)					
No	100/104 (96.2)	34/37 (91.9)	66/67 (98.5)	–^a^	0.13
Yes	4/104 (3.8)	3/37 (8.1)	1/67 (1.5)		
Conversions, no. (%)					
No	97/104 (93.3)	33/37 (89.2)	64/67 (95.5)	–^a^	0.24
Yes	7/104 (6.7)	4/37 (10.8)	3/67 (4.5)		
*Postoperative outcomes*					
Hospital stay, mean (SD), days	8.4 (3.1)	9.4 (3.8)	7.8 (2.5)	2.52	0.01
Complications, no. (%)					
No	98/104 (94.2)	32/37 (86.5)	66/67 (98.5)	–^a^	0.02
Yes	6/104 (5.8)	5/37 (13.5)	1/67 (1.5)		

*CLC* completion of the learning curve

^a^Fisher’s exact test

### Cumulative sum analysis of the length of operation and its components

**Fig. 1:**
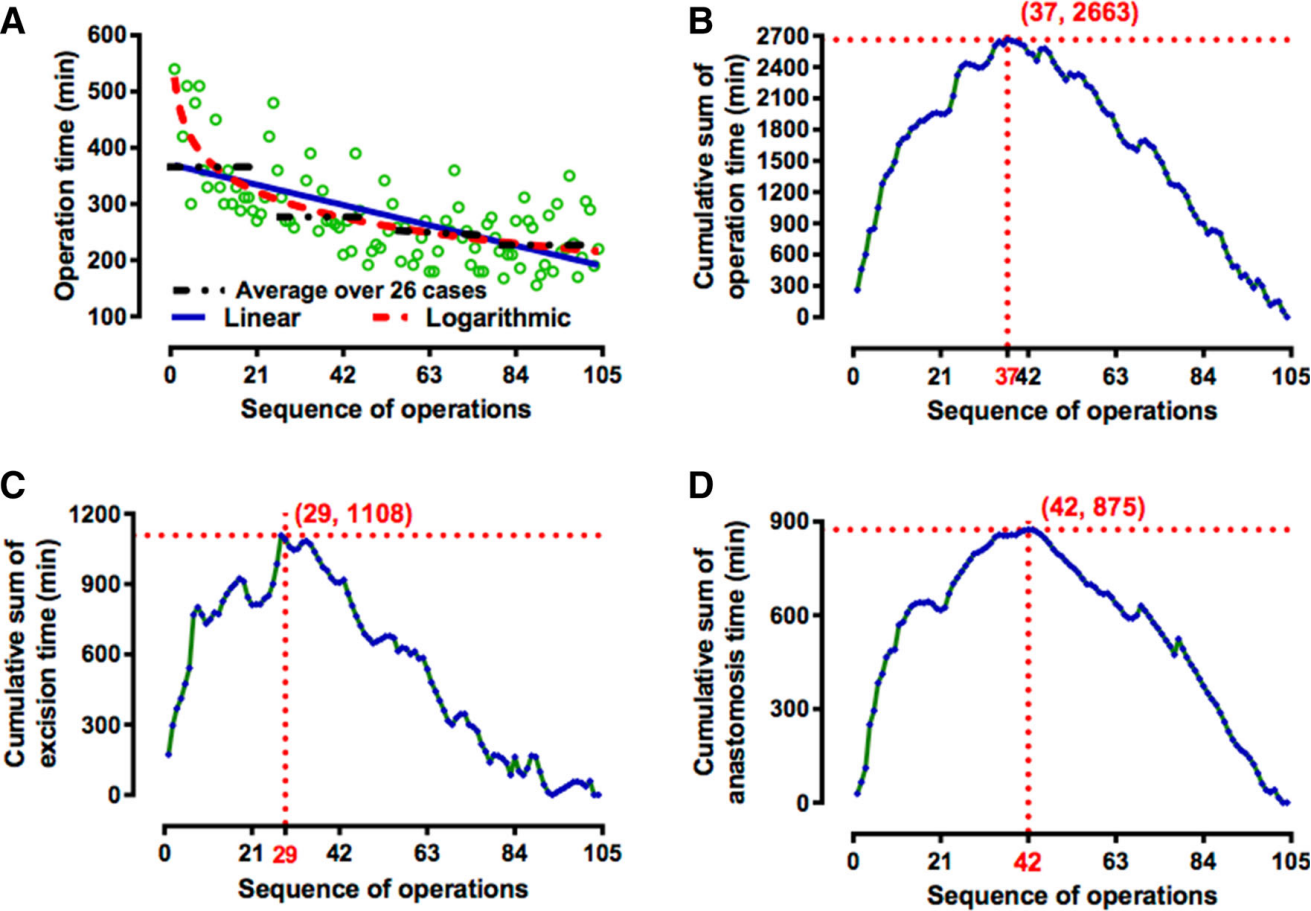
Learning curve of the first 104 consecutive laparoscopic choledochal cyst excision and Roux-en-Y hepaticojejunostomy pediatric cases. *Note* **A** Correlation between the length of the operation and the sequence of the procedures performed, **B** cumulative sum (CUSUM) plot for the overall surgical time, **C** excision time, and **D** anastomosis time

As shown in Fig. 1, the length of the operation ranged from 156 to 540 min with an average of 278 min. The average time for the first 26 cases was 368 min, which improved to 275 min for the next 26 cases and 231 min for the last 26 cases (Fig. 1A). The length of the operation and the consecutive series of procedures presented both a statistically significant logarithmic correlation (*R* ^2^ = 0.55, *P* = 4.0 × 10^−18^) and a significant linear correlation (*R* ^2^ = 0.42, *P* = 1.13 × 10^−12^). However, to reduce the influence of outlying values, it was transformed logarithmically assuming a near-normal distribution.

On visual assessments of the CUSUM plots, a downward inflexion point for decreasing total operating time was observed after patient 37. When specific CUSUM charts resulting from sub-analyses were plotted, however, decreasing excision time was seen after patient 29 and decreasing anastomotic time after patient 42 (Fig. 1).

### Distribution of pre-, intra-, and postoperative factors between the early- and late-experience groups

Using a CLC cutoff of 37 procedures, we divided the 104 patients into two groups: group A: the first 37 patients and group B: the remaining 67 patients. The mean duration of the operation (352.2 ± 80.5 vs. 240.5 ± 50.6 min; *P* < 0.001), the rate of postoperative complications (13.5 vs. 1.5 %; *P* = 0.02), and the length of hospital stay (9.4 ± 3.8 vs. 7.8 ± 2.5 days; *P* = 0.01) were significantly different between the two groups. Stratified analyses revealed that both excision time (*P* < 0.001) and anastomotic time (*P* < 0.001) of group A were significantly longer than that of group B (Table 1).

With regards to other parameters, we found that all the preoperative characteristics (patient-specific factors) (including age, gender, disease duration, and clinical symptoms) and the other intraoperative parameters (procedure-specific factors) measured including blood loss, transfusion rate, adhesion score, and laparotomy conversion rate were similar between the two groups (Table 1).

### The relative impact of key factors on operative time

Multiple linear regression analyses were conducted to determine the relative impact of the key factors on operative time. As shown in Table 2, only the CLC and dense adhesion were independently associated with the length of the operation. The CLC significantly reduced the operating time by 32 % (OR 0.68; 95 % CI 0.63–0.73; *P* < 0.001), and dense adhesion significantly prolonged operating times, as expected (OR_middle_ 1.25; 95 % CI 1.08–1.45; *P* = 0.002, and OR_high_ 1.40; 95 % CI 1.20–1.62; *P* < 0.001).

**Table 2:** Multifactorial analysis of factors associated with logarithm of the length of operation

Variables	*β*	SE	OR	95 % CI	*P*
*Preoperative characteristics*					
Sex (male vs. female)	0.04	0.05	1.04	0.96–1.14	0.35
Age (for each month)	9.87 × 10^−5^	0.001	1.00	0.99–1.00	0.92
Comorbidities (yes vs. no)	0.24	0.17	1.27	0.91–1.77	0.16
Disease duration (vs. ≤30 days)					
1–6 months	−0.05	0.08	0.95	0.81–1.11	0.50
>6 months	−0.07	0.07	0.94	0.82–1.08	0.36
Type of cyst (vs. 1a)					
1c	−0.05	0.04	0.95	0.87–1.04	0.24
4a	−0.04	0.05	0.96	0.86–1.07	0.42
Size of cyst (for each cm)	0.001	0.006	1.00	0.99–1.00	0.85
Abdominal pain (yes vs. no)	−0.01	0.05	0.99	0.90–1.09	0.81
Jaundice (yes vs. no)	0.05	0.05	1.05	0.95–1.15	0.34
Jaundice subsided (yes vs. no)	−0.11	0.07	0.90	0.79–1.03	0.12
CLC (group B vs. group A)	−0.39	0.04	0.68	0.63–0.73	<0.001
*Intraoperative characteristics*					
Adhesions score (vs. low)					
Middle	0.23	0.07	1.25	1.08–1.45	0.002
High	0.33	0.08	1.40	1.20–1.62	<0.001

**Fig. 2:**
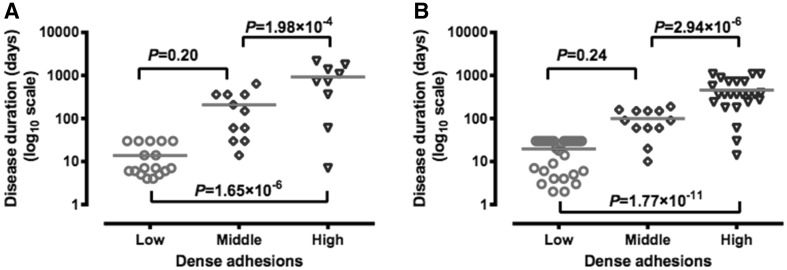
Comparison of the means of disease duration (days) in different levels of adhesion. *Note* **A** group A and **B** group B

The adjusted estimates for the parameter of disease duration, however, were not in complete agreement with univariate analyses, where OR_1–6 months_ 1.10 (95 % CI 0.95–1.28; *P* = 0.22) and OR_>6 months_ 1.24 (95 % CI 1.10–1.39; *P* < 0.001) when compared with those less or equal to 1 month. Specifically, after adjustment for the adhesion score, there was no significant association between disease duration and the length of the operation. ANOVA analyses were used to demonstrate the difference of disease duration among the patients with different extents of adhesion in both group A and group B. The change in disease duration according to status on the adhesion score is illustrated in Fig. 2, and it reveals that the higher the adhesion score, the longer the disease duration.

### Predictors of perioperative adverse outcomes

Overall adverse outcome rate was 18.3 % (19/104), including conversion to open surgery (7 cases, Table 3), transfusion (4 cases), postoperative complications (6 cases, Table 4), wound liquefaction (1 case), and wound dehiscence (1 case). On multivariate analysis, only 2 factors were found to be independent predictors of adverse outcomes: the preoperative comorbidities and the CLC (operative experience) (Table 5). Having adjusted for confounding variables, there was still a 93 % reduction in the likelihood of occurrence of adverse outcomes during the last 67 cases in comparison with the rate following operative experiences within 37 cases. However, patients experiencing one or more comorbidities were 30 times more likely to undergo adverse events than patients without comorbidities (95 % CI 1.71–549.63, *P* = 0.02).

**Table 3:** Characteristics of the patients converted to open surgery

No.	Group	Sex	Age (months)	Symptom	Reasons for conversion
1	1	F	132	Pain with jaundice	Dense adhesion
2	1	F	17	Asymptomatic	Dense adhesion
3	1	F	29	Jaundice	Loop rotation
4	1	F	25	Pain	Hepatic duct stenosis
5	2	F	36	Pain	Accessory hepatic duct
6	2	F	48	Pain	Dense adhesion and capillary hemorrhage
7	2	F	84	Pain	Accompanied with paraduodenal hernia

**Table 4:** Characteristics of the patients with postoperative complications

No.	Group	Sex	Age (months)	Symptom	Complication	Treatment
1	1	F	72	Abdominal pain	Bile leakage	Re-laparotomy and re-anastomosis
2	1	F	36	Abdominal pain	Pancreatic leakage	Conservative treatment for 2 weeks
3	1	F	32	Jaundice	Bile leakage	Conservative treatment for 2 weeks
4	1	M	30	Abdominal pain	Abdominal fluid collection	Conservative treatment
5	1	F	28	Pain and jaundice	Hemoperitoneum	Re-laparotomy
6	2	F	5	Jaundice	Chyloperitoneum	Conservative for 38 days

**Table 5:** Factors influencing the postoperative adverse outcomes

Variables	*β*	SE	OR	95 % CI	*P*
*Preoperative characteristics*					
Sex (male vs. female)	−1.22	1.08	0.29	0.04–2.44	0.26
Age (for each month)	0.003	0.02	1.00	0.97–1.03	0.83
Comorbidities (yes vs. no)	3.42	1.47	30.65	1.71–549.63	0.02
Disease duration (vs. ≤ 30 days)					
1–6 months	0.93	1.37	2.53	0.17–37.35	0.50
>6 months	−0.79	1.70	0.45	0.02–12.69	0.64
Type of cyst (vs. 1a)					
1c	0.73	0.87	2.07	0.37–11.47	0.41
4a	−0.64	0.98	0.53	0.08–3.57	0.51
Size of cyst (for each cm)	−0.01	0.14	0.99	0.76–1.29	0.92
Abdominal pain (yes vs. no)	−0.72	0.87	0.49	0.09–2.71	0.41
Jaundice (yes vs. no)	0.72	1.04	2.06	0.27–15.78	0.49
Jaundice subsided (yes vs. no)	−0.92	1.39	0.40	0.03–6.09	0.51
CLC (group B vs. group A)	−2.64	0.79	0.07	0.02–0.34	0.001
*Intraoperative characteristics*					
Dense adhesions (vs. low)					
Middle	−0.07	1.52	0.93	0.05–18.32	0.96
High	0.69	1.41	1.99	0.13–31.53	0.63

### Factors contributing to a prolonged stay after laparoscopic choledochal cyst excision

All patients were discharged between 5 and 38 days. Multiple linear regression analysis among patients receiving laparoscopic choledochal cyst excision showed that, after adjustment, preoperative comorbidities and perioperative adverse events were associated with a significantly prolonged postoperative stay (Table 6). Patients with comorbidities and adverse outcomes stayed 123 and 33 % longer, respectively, comparing with patients without these events. The CLC was also a significant predictor. Postoperative stay of late-experience group was 14 % shorter than that of early group.

**Table 6:** Characteristics associated with logarithm of the length of postoperative hospital stay

Variables	*β*	SE	OR	95 % CI	*P*
*Preoperative characteristics*					
Sex (male vs. female)	−0.10	0.09	0.90	0.76–1.07	0.25
Age (for each month)	−0.001	0.002	1.00	0.98–1.03	0.42
Comorbidities (yes vs. no)	0.80	0.19	2.23	1.55–3.21	<0.001
Disease duration (vs. ≤30 days)					
1–6 months	−0.23	0.15	0.80	0.59–1.07	0.13
>6 months	−0.02	0.13	0.98	0.75–1.27	0.88
Type of cyst (vs. 1a)					
1c	−0.08	0.08	0.92	0.78–1.09	0.33
4a	−0.18	0.09	0.84	0.70–1.00	0.06
Size of cyst (for each cm)	0.002	0.01	1.00	0.98–1.03	0.84
Abdominal pain (yes vs. no)	0.05	0.09	1.05	0.89–1.25	0.56
Jaundice (yes vs. no)	0.21	0.07	1.23	1.07–1.43	0.005
Jaundice subsided (yes vs. no)	0.17	0.12	1.19	0.93–1.50	0.16
CLC (group B vs. group A)	−0.15	0.07	0.86	0.76–0.98	0.03
*Intraoperative characteristics*					
Dense adhesions (vs. low)					
Middle	0.17	0.14	1.19	0.90–1.57	0.22
High	−0.05	0.14	0.95	0.73–1.26	0.74
*Perioperative adverse outcomes*					
Adverse outcomes (yes vs. no)	0.28	0.09	1.33	1.11–1.59	0.002

## Discussion

Up to now, there has been no formal analysis for learning curve of laparoscopic choledochal cyst excision and Roux-en-Y hepaticojejunostomy. To the best of our knowledge, this is the first report in children. The learning curve refers to the course of mastering a particular procedure through continuous practice [14]. The initial training period or learning curve represents the rapid change in the ability to complete the task until “failure” is eliminated or reduced to a minimum constant rate. There are many methods to evaluate the learning curve. The simple ones use simple graphs, arbitrarily splitting of the data into chronologic groups and performing univariate statistics with and without tests for trend [15, 16]. There are some shortcomings about these methods. Cumulative sum (CUSUM) analysis transforms raw data into the running total of data deviations from their group mean, enabling the visualization of trends in a dataset, which is different from other approaches [17]. In this study, the CUSUM analysis was used to obtain more forceful results of the learning curve for CDC.

Different surgical procedures have different lengths of CLC, i.e., CLC for laparoscopic cholecystectomy is 20 cases [18] and for distal pancreatic tail resection is 17 cases [19]. The laparoscopic choledochal cyst surgery is a procedure with more technical challenge and complexity. It requires a longer time to complete the training. In this study, we found that the CLC was approximately 37 cases, which is more than many other procedures. Our result is similar to Diao’s [6] report, in which the CLC is estimated as 35 cases. But her result is based on the comparison of the operative time without any statistical tests for trend. In our study, the CUSUM analysis gives us a clear view about the trend of the operative time. The analysis of the dataset supports the significance of the cutoff point of the learning curve. The results show that, after the CLC, not only the operative time is reduced, the complications, adverse results, and length of hospital stay also decreased significantly. The patients have better results after CLC.

Resection of choledochal cyst and the hepaticojejunostomy are the two major and most difficult steps in this laparoscopic procedure [20]. In order to understand the features of the learning curve better, the resection time and the anastomosis time were calculated, respectively, with the CLC of 29 cases and 42 cases, respectively. Combining the resection time and the anastomosis time, the completion of the whole learning curve is about 37 cases based on the single operator’s experience. This reflects the different features of the two courses in the operation. Anastomosis is a more difficult technique to master in laparoscopic surgery. Once the anastomosis skill is mastered, bile leakage resulting from the hepaticojejunostomy can be reduced or completely avoided. However, the anastomosis time can only be shortened after extensive practice. By the end of the study, the anastomosis time is 20–30 min, and the shortest one is 17 min. In contrast to the anastomosis technique, the excision technique is easier to be mastered, especially in patients with less adhesion.

There are many factors related to the operative time of laparoscopic choledochal cyst excision and hepaticojejunostomy. In the present study, only the CLC and the extent of adhesion were independently associated with the length of the operation. It means that the adhesion is the second most influential factor to the operative time after CLC, either before or after the completion of the training. The adhesion differs much among patients, and the length of the dissection time will also differ a lot according to condition of the patients. The dissection time is the most variable factor in the operation. So it is not surprising that even after the completing the learning curve, adhesion still require prolonged operative time.

The pathologic change of choledochal cyst depends on the duration and the severity of the pathology. With the progress of the disease, the mucosa of the cyst is damaged or even disappeared, the cystic wall become thickened, small vessels develop on the surface of the cyst, and more adhesions develop between the choledochal cyst and surrounding vital structures, such as portal vein and hepatic artery [21]. In the present study, adhesion surrounded the cyst is associated with longer disease history. This leads to more difficulty in dissection and a longer operative time. It was previously assumed that the older the patients, the denser the adhesion. We found that the duration of the active disease rather than the age of the patients correlates with the adhesion score. Clinically, we often find that in older children with no history of infection, the adhesion was not severe. However, occasional mild attacks may be ignored by parents. This makes it difficult to assess the exact duration of active infection to predict the degree of adhesion preoperatively. For surgeons in the early learning curve, we suggest patient selection criteria as follows: It is better to select easy case with short disease history, so as to accomplish the operation safely and uneventfully, in another word, avoid the patients with long disease history, or older children whose history cannot be determined definitely.

There was no intraoperative complication in our study. The postoperative complications were encountered in six children. The occurrence is similar to many previous reports. In Liem’s series [8] of 309 cases of laparoscopic excision of choledochal cyst, the complication rate is 11/309. Of the six complications in our study, five was in group A and one in group B. In group A, biliary leakage occurred in two patients, one required an open revision of hepaticojejunostomy, and the other one resolved with medical treatment 2 weeks after the primary operation. Pancreatic leakage occurred in one patient, which resolved spontaneously. Hemoperitoneum was found in one patient on the day 1, open laparotomy was performed immediately, and it is disclosed that the hemorrhage is from the site of the trocar port. And there was also an abdominal fluid collection, which settled spontaneously. All of the complications above occurred in group A are more likely due to poor surgical techniques. The laparoscopic surgery is technique demanding. Hepaticojejunostomy, which is an advanced technique, is related to the two bile leakages in our study. The proper layer between the cyst and the pancreas is the key point for cyst dissection, but it may be obscure in some dense adhesion cases. The reason for the pancreatic leakage in group A is probably because of the damage to pancreatic tissue, where the adhesion is quite severe between the cyst and pancreas. But with more experience and improved technique, with a finer anastomosis and a meticulous dissection, such complications have been reduced or avoided. Fortunately, there is no bile leak or pancreatic leak in the subsequent surgery. After the initial period, we think routine placement of abdominal drain is not necessary. When the cyst dissection is easy to finish with little exudate and the anastomosis is satisfied, the abdominal drain would be omitted. In case of dense adhesion around the cyst and a lot of exudate after dissection, or when a ductoplasty is performed, the abdominal drain would be placed. The only complication in group B was chyloperitoneum, which was found 5 days postoperatively, following oral feeding. With conservative treatment, the ascites healed spontaneously and the patient discharged 38 days later.

The conversion rate in this study is also similar to other reports [22, 23]. Seven operations were converted to open surgery, with 4 in group A and 3 in group B, with no significant difference. The reasons for the conversion were shown in Table 3. In group A, the reasons for conversion are: severe adhesions in 2 cases, twisting of RY limb in one, and difficulty to accomplish ductoplasty in one. All of them seemed to be due to technical reasons. In group B, the causes of conversion are: one severe adhesion, one of injury of the accessory bile duct, and one of complicated comorbidity of paraduodenal hernia. The injury of the accessory bile duct led to conversion to open surgery to accomplish the cholangiojejunal loop anastomosis. As the technique improved, the conversion resulted from the technical reasons reduces. Hence, it is not surprising that there is no conversion in the last 41 cases in this study.

The long operative time in the early cases is mainly due to caution and logistic problems. As we accumulate operative experience and improve our laparoscopic techniques, the operative time will be shortened and the complications decreased. Some technical maneuvers can facilitate manipulations, such as: the traction suture and bipolar cautery hook. The traction suture can be placed on the round ligament to elevate the liver and enlarge the operative field of the liver hilar area, be placed on the front wall of the common hepatic duct to facilitate the hepaticojejunostomy, or be placed on the front wall of the choledochal cyst to facilitate the dissection of the distal end of the common bile duct. When dense adhesion is encountered, the bipolar cautery hook is an effective instrument for dissection to reach the proper layer and to achieve hemostasis. For huge cysts, it is better to incise the anterior wall to decompress the cyst to facilitate the dissection. All these operative tips are useful to complete the surgery smoothly and time-saving.

Although the operative time is a good parameter for measurement of the learning curve, the adverse events, such as conversion and postoperative complication, are the most important measurement to CLC and the result of the operation. Only when the adverse events decreased significantly to a satisfied level, is the learning curve completed. From the analyses of our results, it seems that most adverse events are due to technical reasons, such as bile leakage, pancreatic leakage, and difficulty in dissection. So, we think the laparoscopic skill is a crucial factor in the completion of the learning curve. Minimal invasive surgery training and simulation are essential to obtain the technical skills. Experience from other laparoscopic procedures is also helpful. Besides, supervision by an experienced surgeon would also be necessary in reducing adverse events in the learning curve period. All these methods should be useful to shorten CLC, especially in lower-volume centers. The MIS training program based on learning curve would be a subject to be studied.

However, our study had significant limitations. Clearly, the learning curve varies with the frequency in which patients are operated on, the type and volume of the practice, and many parameters peculiar to the individual surgeon. The present study only represents the experience of a single surgeon. The training and inherent skill is different among individual surgeons. So, these findings cannot be applied to all surgeons or clinical settings. However, this study provides a reasonable reference of learning curve for other surgeons.

## Conclusions

The learning curve for the laparoscopic excision of CDC and Roux-en-Y hepaticojejunostomy in children is 37 cases. After completing the learning curve, the surgeon-specific outcomes significantly improved in terms of operative time, overall postoperative complication rate, and the length of hospital stay. The learning curves for the treatment of CDC can be used as the basis for performance guiding the training and implementation at institutions not currently using this technique.
